# CD-NP: A Novel Engineered Dual Guanylyl Cyclase Activator with Anti-Fibrotic Actions in the Heart

**DOI:** 10.1371/journal.pone.0052422

**Published:** 2012-12-18

**Authors:** Fernando L. Martin, S. Jeson Sangaralingham, Brenda K. Huntley, Paul M. McKie, Tomoko Ichiki, Horng H. Chen, Josef Korinek, Gerald E. Harders, John C. Burnett

**Affiliations:** Cardiorenal Research Laboratory, Division of Cardiovascular Diseases, Mayo Clinic, Rochester, Minnesota, United States of America; University of Otago, New Zealand

## Abstract

Natriuretic peptides (NPs) are cardioprotective through the activation of guanylyl cyclase (GC) receptors A and B. CD-NP, also known as cenderitide, is a novel engineered NP that was designed to uniquely serve as a first-in-class dual GC receptor agonist. Recognizing the aldosterone suppressing actions of GC-A activation and the potent inhibitory actions on collagen synthesis and fibroblast proliferation through GC-B activation, the current study was designed to establish the anti-fibrotic actions of CD-NP, administered subcutaneously, in an experimental rat model of early cardiac fibrosis induced by unilateral nephrectomy (UNX). Our results demonstrate that a two week subcutaneous infusion of CD-NP significantly suppresses left ventricular fibrosis and circulating aldosterone, while preserving both systolic and diastolic function, in UNX rats compared to vehicle treated UNX rats. Additionally we also confirmed, *in vitro*, that CD-NP significantly generates the second messenger, cGMP, through both the GC-A and GC-B receptors. Taken together, this novel dual GC receptor activator may represent an innovative anti-fibrotic therapeutic agent.

## Introduction

CD-NP (cenderitide) is a new designer natriuretic peptide (NP) that has been engineered with the ability to co-activate the two known NP receptors, guanylyl cyclase (GC) -A and -B. Unlike ANP and BNP, which activate the GC-A receptor [1], [2], or CNP which has high affinity for the GC-B receptor [3], [4], CD-NP has been reported in one study to activate both GC receptors [5] and consequently activating the second messenger, cyclic 3′,5′ guanosine monophosphate (cGMP). Importantly CD-NP, like CNP, activates cGMP to a greater magnitude in human cardiac fibroblasts (hCFs) than the other GC-A activators such as BNP and inhibits hCF proliferation induced by the potent pro-fibrotic cytokine cardiotrophin-1 [6]. Furthermore, Horio and colleagues have established that CNP is the most potent fibro-inhibiting of the native NPs [7]. However the therapeutic use of CNP has being limited due to its rapid degradation in the circulation by neutral endopeptidase [8].

**Figure 1:**
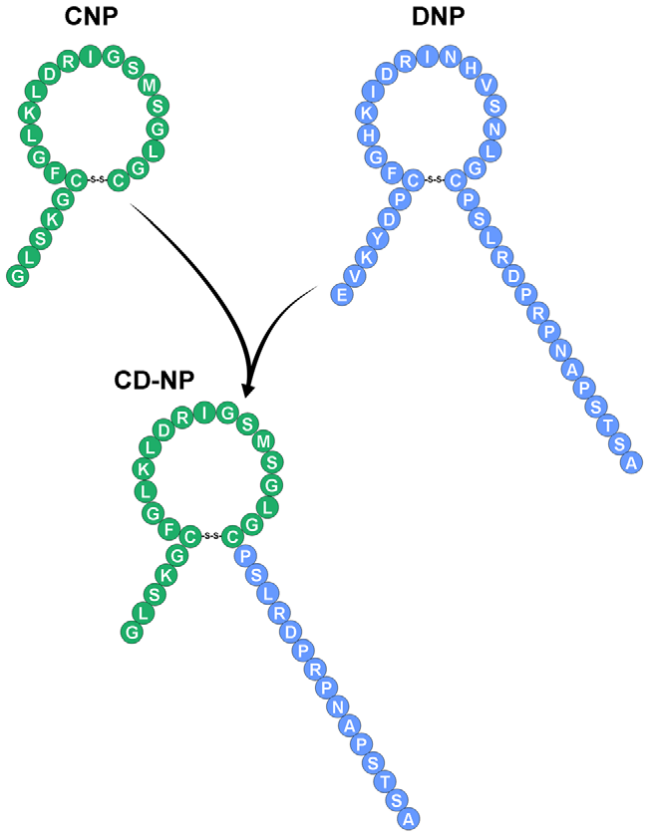
Natriuretic Peptide Structures. Amino acid sequence of mature C-type natriuretic peptide (CNP), Dendroaspis natriuretic peptide (DNP) and the designer natriuretic peptide, CD-NP.

The design of CD-NP involves the fusion of human mature CNP with the C-terminus of Dendroaspis natriuretic peptide (DNP), which was first isolated from the venom of the green mamba [9] (Figure 1). DNP is a potent GC-A agonist [5] and is also highly resistant to enzymatic degradation compared to the native NPs, due to its 15 amino acid (AA) C-terminus [8]. We initially hypothesized that fusing the C-terminus of DNP to mature CNP would not only render CD-NP more resistant to degradation than CNP, but also would result in a dual GC-A and GC-B activator that retains the biological actions of both receptors including the anti-proliferative and anti-fibrotic properties of CNP via the GC-B receptor and the aldosterone suppressive properties of DNP via the GC-A receptor. Importantly a first in human clinical trial demonstrated that CD-NP, in normal volunteers, reduced circulating aldosterone while placebo did not [10].

Thus, the *in vitro* and *in vivo* properties of CD-NP support its potential as a novel anti-fibrotic therapeutic drug through dual GC receptor activation such as inhibition of collagen deposition as well as inhibition of aldosterone. While previous investigations have reported a direct link in CNP's ability to attenuate organ fibrosis in experimental models of disease [11], [12], the *in vivo* anti-fibrotic properties of CD-NP have not been addressed to date. Therefore, the current study was designed with two main objectives. First, we sought to confirm and extend previous reports that CD-NP co-activates the GC-A and GC-B receptor *in vitro*. Here we measured cGMP generation in human embryonic kidney cells (HEK), transfected with human GC-A or GC-B receptor, after stimulation with a pharmacological dose of CNP, DNP and CD-NP for 10 minutes. Our second objective was to determine the ability of chronic subcutaneously (SubQ) delivered CD-NP to prevent the development of cardiac fibrosis in an experimental rat model of mild renal insufficiency induced by unilateral nephrectomy (UNX), which we have recently reported as a model that mediates early cardiac fibrosis and diastolic dysfunction [13]. Here we administered CD-NP, SubQ, for two weeks beginning at the time of UNX. To assess the potential of sustained actions of CD-NP upon the prevention of cardiac fibrosis and diastolic impairment, the heart was assessed by echocardiography two weeks after discontinuation of CD-NP infusion, after which cardiac fibrosis was quantified and circulating aldosterone was determined. We hypothesized that chronic SubQ delivery of CD-NP for two weeks would prevent cardiac fibrosis, preserve diastolic function and maintain normal levels of aldosterone at 4 weeks following UNX.

## Methods

### Peptides and Reagents

CD-NP (Nile Therapeutics, San Mateo CA), CNP and DNP (Phoenix Pharmaceuticals, Burlingame, CA) were used. Structures were confirmed by mass spectrometry, and high-performance liquid chromatography analysis confirmed purity to be >95%. Human GC-A and GC-B receptor cDNA clones were purchased from Origene (Rockville, MD).

### Cell Stimulation Studies and cGMP Assay

Human embryonic kidney (HEK) 293 cells were stably transfected with either the human GC-A or GC-B receptor using Lipofectamine (Invitrogen, Grand Island, NY). Transfected cells were maintained in Dulbecco's modified Eagle's medium supplemented with 10% fetal bovine serum, 100 U/ml penicillin, 100 U/ml streptomycin, and 250 ug/ml G418 (all reagents from Invitrogen, Grand Island, NY). HEK cells were plated in 6-well plates and treated as previously described [14] Briefly, cells were incubated in Hank's balanced salt solution (Invitrogen, Carlsbad, CA) containing 20 mmol/L N-[2-hydroxyethyl]piperazine-N'[2-ethanesulfonic acid], 0.1% bovine serum albumin, and 0.5 mmol/L 3-isobutyl-1-methylzanthine (Sigma, St. Louis, MO). Treated cells received 10^−6^ M of CD-NP, CNP or DNP for 10 minutes. Cells were lysed in 300 ul 6% TCA and sonicated for 10 min. The samples were ether extracted four times in 4 volumes of ether, dried, and reconstituted in 300 μl cGMP assay buffer. The samples were assayed using a competitive radioimmunoassay (RIA) cGMP kit (Perkin-Elmer, Boston, MA) as previously described [15]. Samples are corrected for dilution factors and protein concentration, and values are expressed as pmol/well. There is no cross-reactivity with ANP, BNP, CNP, ET, and <0.001% cross-reactivity with cAMP, GMP, GDP, ATP, GTP.

### Animals and Ethics

Twenty-four male Wistar rats (Charles River Laboratories, Wilmington, MA) approximately 8 weeks old and weighing 250 g were used. The experimental study conformed to the Guide for Humane Care and Use of Laboratory Animals published by the National Institutes of Health (Public Health Service Approved Animal Welfare Assurance number: A3291-01). All animal usage, experimental procedures and protocols were reviewed and approved by the Institutional Animal Care and Use Committee at Mayo Clinic.

### Experimental Groups

Three groups were used: Group 1: SHAM+Vehicle (5% glucose solution; n = 8); Group 2: UNX+Vehicle (5% glucose solution; n = 8); Group 3: UNX+ CD-NP (170 ng/kg/min of CD-NP was dissolved in 5% glucose solution; n = 8). An equimolar dose of CD-NP was calculated and used based on a previous published study using CNP [12]. On day one rats were randomly assigned to the three different groups.

### Surgical Procedure

**Figure 2:**
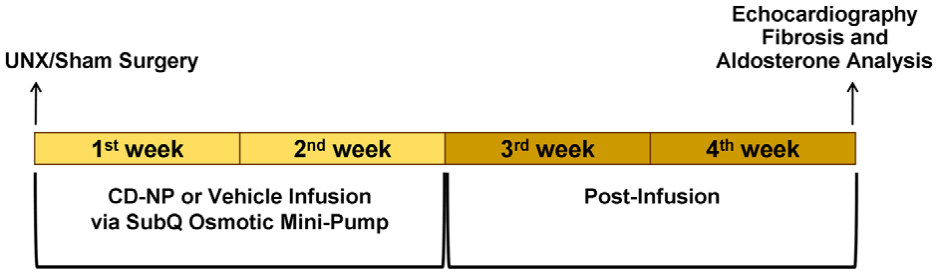
Study Timeline. Experimental protocol of CD-NP (170 ng/kg/min in 5% glucose solution) or Vehicle (5% glucose solution) delivery in Sham or UNX-operated male Wistar rats by subcutaneous (SubQ) osmotic mini-pump.

A laparotomy was performed on anesthetized (1.5% isoflurane in oxygen) rats and the whole right kidney was removed in UNX rats as previously described [13]. Rats in the Sham group underwent the same procedure but no kidney was removed. Vehicle (5% glucose solution) or CD-NP (170 ng/kg/min in 5% glucose solution) treatment was administered through a SubQ implanted osmotic mini-pump (Alzet Model 2ML1, Alzet, Durect Corporation, Cupertino, CA). A small incision was made in the skin between the scapulae. Using a hemostat, a small pocket was formed by spreading the subcutaneous tissues apart. The pump was inserted into the pocket with the flow pointing away from the incision. The skin incision was closed using staple clips. The osmotic mini-pump was inserted and treatment was initiated at the time of Sham or UNX surgery. The infusion of CD-NP or Vehicle was maintained for two weeks. The experimental protocol (Figure 2) was continued for another two weeks, without CD-NP or Vehicle infusion, for a total of four weeks.

### Echocardiography

At 4 weeks post-surgery, standard transthoracic echocardiography was performed on anesthetized (1.5% isoflurane in oxygen) rats using the Vivid 7 ultrasound system (GE Medical Systems, Milwaukee, WI) and a 10S transducer (11.5 MHz) with ECG monitoring as previously described [13], [16], [17]. Briefly, M-mode images and gray scale 2D parasternal short axis images (300–350 frames/sec) at the mid-papillary level were recorded for off-line analysis using EchoPAC software (EchoPAC PC BTO 9.0.0, GE Healthcare, Milwaukee, WI). LV ejection fraction (EF) was calculated from M-mode image measurements. Two-dimensional speckle derived strain echocardiography parasternal short axis images at the mid-papillary level were acquired with a frame rate ranging from 60 (full apical views) and 160 (narrow sector views) frames/sec. Three consecutive cardiac cycles were recorded as 2-D cine loops and the acquired raw data were saved for off-line analysis. Speckle tracking was performed by the EchoPAC software and global circumferential strain and strain rates parameters were measured. The analysis included peak circumferential contraction strain (sS) and strain rates (sSR) for evaluation of myocardial systolic function and peak early (dSR-E) and late relaxation (i.e. atrial contraction, dSR-A) circumferential strain rates and their ratio for evaluation of myocardial diastolic function. All parameters represent the average of 3 beats.

### Blood Pressure and Plasma Aldosterone Analysis

After echocardiography, PE-50 tubing was placed into the carotid artery for blood pressure (BP) monitoring and blood sampling. After BP acquisition using CardioSOFT Pro software (Sonometrics Corporation, London, Ontario), blood was collected from the carotid artery and placed in EDTA tubes on ice. Blood was immediately centrifuged at 2,500 rpm at 4°C for 10 minutes and the plasma was stored in polystyrene tubes at −80°C. Plasma aldosterone was determined by a commercially available RIA as described previously [18].

### Left Ventricular Tissue Harvest and Fibrosis Analysis

After blood collection, hearts were removed for total cardiac and left ventricular (LV) weights. A mid-LV cross-section was preserved in 10% formalin for fibrosis analysis. As previously described [17], fixed LV tissues were dehydrated, embedded in paraffin and sectioned at thickness of 4 µm. Fibrillar LV interstitial collagen and extent of fibrosis was performed using picrosirius red staining. An Axioplan II KS 400 microscope (Carl Zeiss, Inc., Gottingen, Germany) was used to capture at least 4 randomly selected LV images, between the sub-epicardial and sub-endocardial regions, from each slide using a 10x objective magnification. KS 400 software was utilized to determined fibrotic area as a percentage of total tissue area.

### Statistical Analysis

Descriptive statistics are reported as mean ± SEM. Unpaired t-test was performed for comparison between *in vitro* groups, while comparisons within *in vivo* groups were made by 1-way analysis of variance (ANOVA) followed by Newman-Keuls post test analysis. GraphPad Prism 5 (GraphPad Software, La Jolla, CA) was used for the above calculations. Statistical significance was accepted as *P*<0.05.

## Results

### *In Vitro* cGMP Generation

**Figure 3:**
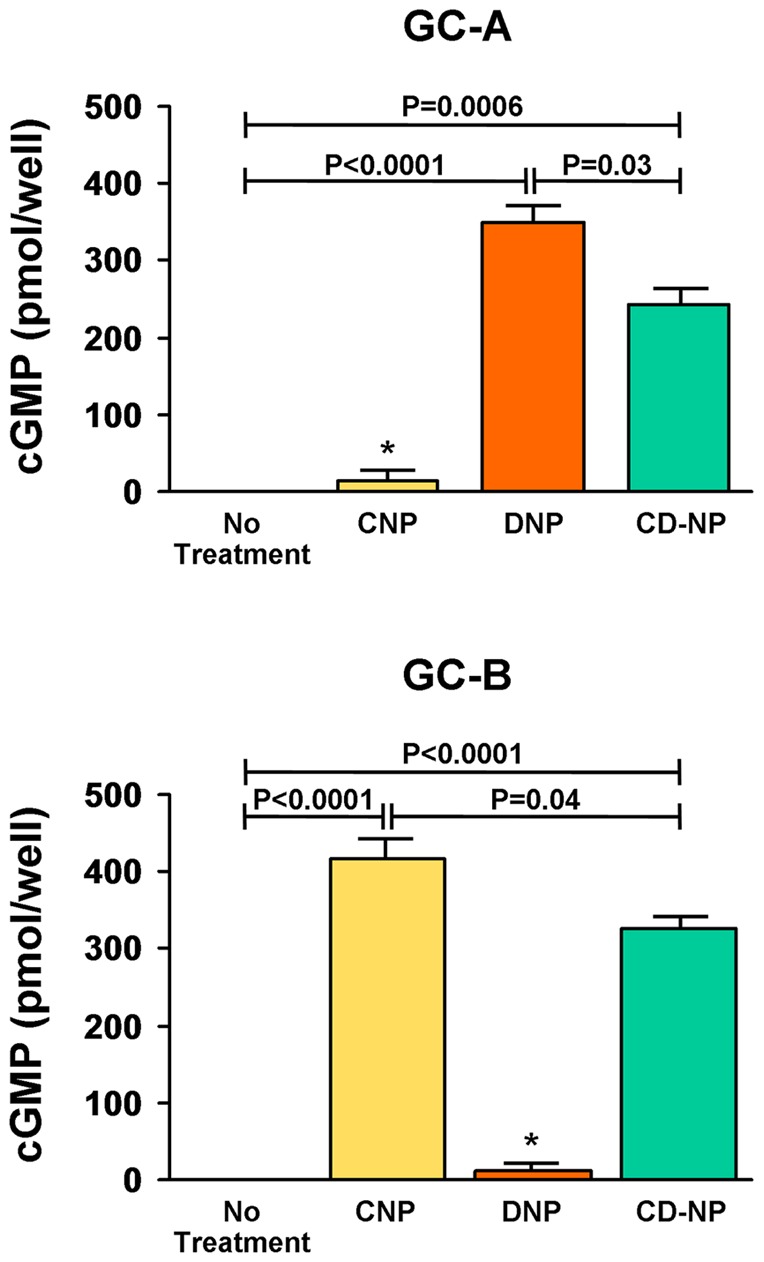
Natriuretic Peptide Generation of cGMP. *In vitro* 3′,5′-cyclic guanosine monophosphate (cGMP) generation in human embryonic kidney 293 cells stably transfected with either the GC-A (upper graph) or GC-B (lower graph) receptor in response to a 10^−6^ M dose of CNP (yellow bar), DNP (orange bar) and CD-NP (green bar) compared with no treatment. Values are mean ± SEM. *P<0.05 vs no treatment.

To determine the ability of various NPs to generate cGMP through either the human GC-A or GC-B receptor, we stably expressed each GC receptor in HEK 293 cells devoid of endogenous GC receptors. Cells were treated with a pharmacological dose (10^−6^ M) of CNP, DNP or CD-NP and compared to no treatment (Figure 3). Both DNP and CD-NP significantly activated cGMP via the GC-A receptor, while DNP had greater cGMP activation (P<0.0001) than CD-NP (P = 0.0006) compared to no treatment. Meanwhile, CNP and CD-NP significantly activated cGMP (P<0.0001) via the GC-B receptor compared to no treatment. Thus, the addition of the C-terminus of DNP to mature CNP resulted in a designer NP that has the ability to co-activate both the GC-A and GC-B receptor at a pharmacological dose.

### *In Vivo* Cardiovascular Structure and Function

**Figure 4:**
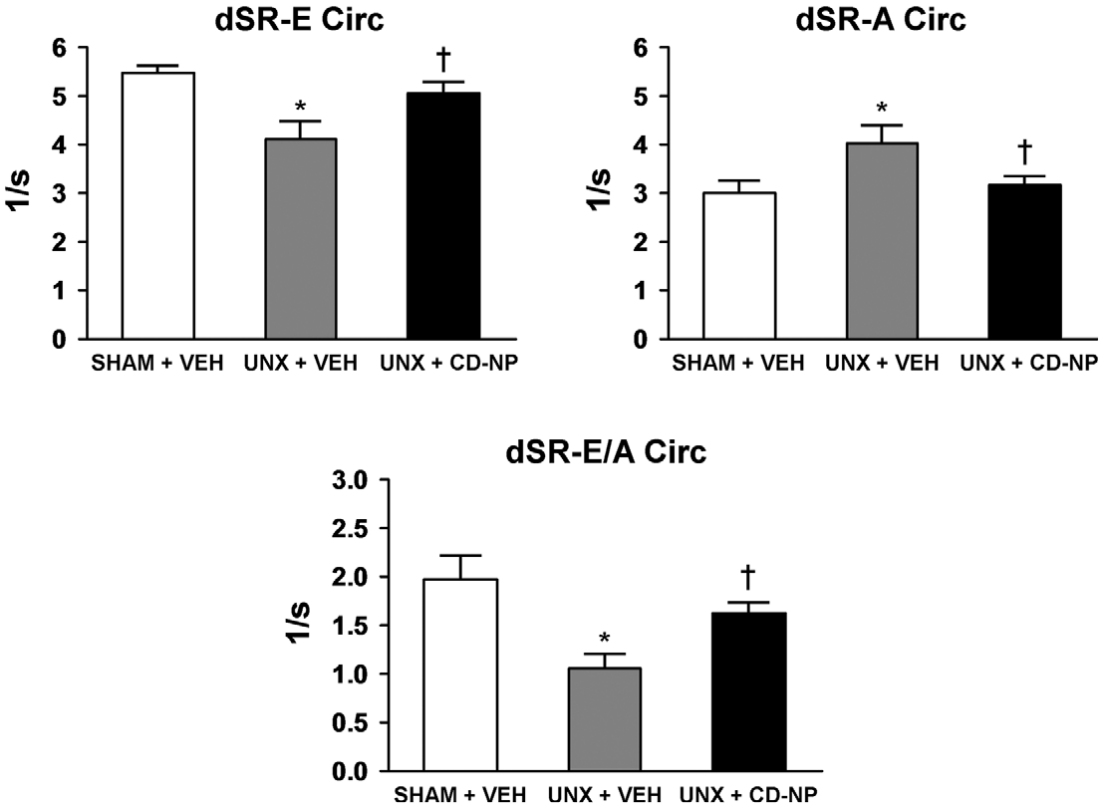
Diastolic Function. Effect of CD-NP treatment on preserving diastolic function in UNX+CD-NP rats compared to UNX+Vehicle rats. Values are mean ± SEM. *P<0.05 vs Sham+Vehicle and 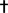 P<0.05 vs UNX+Vehicle.

Cardiac structure, systolic function and BP, in the three experimental treatment groups, are reported in Table 1. Body weight (BW), heart weight (HW), LV weight (LVW), HW normalized to BW and LVW normalized to BW did not differ between the three experimental groups. Further, systolic function as demonstrated by LV EF, circumferential sS and sSR was also the same between the groups. However, diastolic function (Figure 4) as determined by circumferential dSR-E, dSR-A and dSR-E/A was significantly impaired in the UNX+Vehicle rats compared to Sham+Vehicle and UNX+CD-NP treated rats. There was no change in HR or MAP among the groups.

**Table 1:** Cardiac Structure, Systolic Function and Blood Pressure.

	SHAM+VEH (n = 8)	UNX+VEH (n = 8)	UNX+CD-NP (n = 8)
Body Weight (g)	360±10	377±7	373±8
Heart Weight (mg)	1084±37	1035±20	1047±44
HW:BW (mg/g)	2.94±0.08	2.80±0.07	2.90±0.05
LV Weight (mg)	738±47	731±21	738±28
LVW:BW (mg/g)	2.09±0.05	2.06±0.05	2.08±0.05
HR (bpm)	374±7	395±15	388±11
EF (%)	78±1	78±1	81±1
sS Circ (%)	−17.4±0.6	−17.0±0.4	−16.1±0.4
sSR Circ (1/s)	−4.4±0.2	−4.3±0.2	−4.3±0.2
MAP (mmHg)	89±1	91±3	91±2

Values are mean ± SEM. BW  =  body weight; HW  =  heart weight; LV  =  left ventricle; LVW  =  left ventricle weight; HR  =  heart rate; EF  =  ejection fraction; sS  =  systolic strain; sSR  =  systolic strain rate; Circ  =  circumferential; MAP  =  mean arterial pressure.

### LV Fibrosis and Plasma Aldosterone

**Figure 5:**
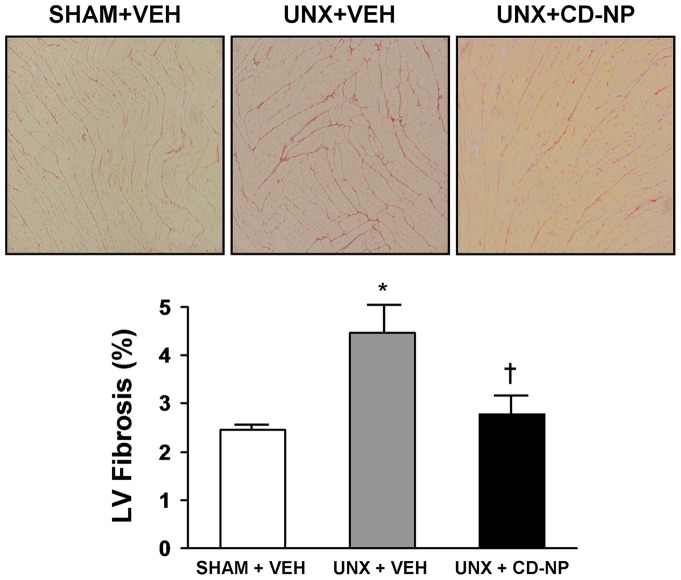
LV Fibrosis. Representative histology images at 10x objective magnification of the LV stained with picrosirius red (upper panels) and the quantification of LV fibrosis staining (lower graph) in Sham+Vehicle, UNX+Vehicle and UNX+CD-NP rats. Values are mean ± SEM. *P<0.05 vs Sham+Vehicle and 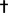 P<0.05 vs UNX+Vehicle.

**Figure 6:**
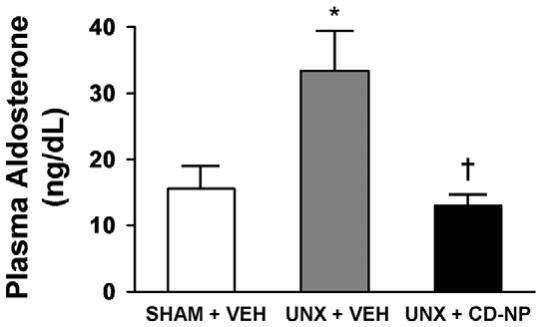
Plasma Aldosterone . Effect of CD-NP treatment in suppressing plasma aldosterone in UNX+CD-NP rats compared to UNX+Vehicle rats. Values are mean ± SEM. *P<0.05 vs Sham+Vehicle and 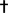 P<0.05 vs UNX+Vehicle.

Figure 5 illustrates a representative image of LV fibrosis using picro-sirius red staining (upper panels) and the quantification of this histological staining (lower graph). Specifically we observed a mild, yet significant increase in the intensity of fibrillar collagen staining in the UNX+Vehicle rats compared to the other two groups. The two week treatment using CD-NP, initiated at the time of UNX, had a sustained and significant effect in attenuating LV fibrosis after UNX. Importantly, circulating aldosterone (Figure 6) was significantly elevated in the UNX+Vehicle rats compared to Sham+Vehicle rats and CD-NP treatment elicited a sustained attenuation of plasma aldosterone in UNX rats maintaining levels which were not different from the Sham+Vehicle rats.

## Discussion

CD-NP (cenderitide) is a novel Mayo Clinic developed designer NP now beginning Phase II clinical trials for chronic therapy in post acute heart failure patients. In the current study we first confirmed that CD-NP, at a pharmacological dose, represents the first and only dual GC receptor activator to date. We also demonstrated for the first time, that SubQ administration of CD-NP has inhibitory effects on LV fibrosis including the suppression of plasma aldosterone in an experimental rat model of early cardiac fibrosis induced by UNX. Importantly, a continuous infusion of CD-NP for two weeks, initiated at the time of a reduction in renal mass via UNX, had sustained anti-fibrotic effects on the heart and preserved diastolic function at four weeks. These findings lay the foundation for future studies aimed at understanding the anti-fibrotic mechanisms of chronic CD-NP therapy in a variety of models of fibrotic disease.

The importance CNP in the regulation extracellular matrix and fibrotic pathways has been underscored by a number of *in vitro* [7] and *in vivo* [11], [12], [16], [19], [20], [21], [22] studies. However, the therapeutic application of CNP remains challenging as CNP is the most susceptible of the NPs to enzymatic degradation [23] and therefore has a short biological half-life [24]. Notably, CD-NP represents a next generation CNP based therapeutic, which consists of addition of a C-terminus to mature CNP (Figure 1). Previous studies have reported that the addition of the C-terminus of DNP to the empty C-terminus of CNP creates a peptide that is highly resistant to degradation by neutral endopeptidase *in vitro* [8]. Furthermore, this C-terminus addition from a specific GC-A activator (i.e. DNP) to CNP, which is a specific GC-B activator, also created the first dual GC receptor agonist known to date [5]. As a result, CD-NP may be a unique NP with high bioavailability that could exploit the beneficial properties of not only one GC receptor, but both, simultaneously. Our result confirms the one previous report by Dickey et al. [5] and clearly establishes that a pharmacological dose of CD-NP activates both the GC-A and GC-B receptor and significantly generates cGMP *in vitro*.

Importantly, the current study represents the first pharmacological report of CD-NP's anti-fibrotic effects *in vivo*. Our previous report established that mild, yet significant LV fibrosis and diastolic impairment was induced at four weeks after UNX [13]. While the exact mechanisms by which UNX induces cardiac fibrosis remains unknown, one might speculate that mechanisms for an increase in LV fibrosis are multi-factorial including an elevation in circulating factors such as aldosterone that contribute to collagen accumulation [25]. In addition, we have previously reported that UNX is associated with cardiomyocyte apoptosis [13] which may be induced by the pro-apoptotic actions of aldosterone [26], [27] or by other mechanisms which remain to be elucidated. Therefore, myocyte death may also contribute to replacement fibrosis.

Nonetheless, using this experimental model of cardiac fibrosis, we have demonstrated that a two week SubQ infusion of CD-NP significantly inhibited and also had sustained effects in attenuating LV fibrosis and preserving diastolic function. This attenuation of LV fibrosis is likely due to diverse mechanisms through activation of both the GC-A and GC-B receptors. Such mechanisms include 1) GC-A mediated suppression of the potent pro-fibrotic factor aldosterone, which has been increasingly noted to induce cardiac fibrosis [25], [28], [29], [30] and 2) a reduction in cardiomyocyte apoptosis via GC-A activation and the subsequent inhibition of aldosterone secretion or through the nuclear accumulation of zyxin and activated Akt [31]. Other possible mechanisms that may also contribute to the prevention of UNX-induced myocardial fibrosis include inhibition of fibroblast proliferation which may be linked to CNP and GC-B activation, given the predominance of GC-B receptors on cardiac fibroblasts [32] and indeed our previous report demonstrated that CD-NP suppresses hCF proliferation *in vitro* [6]. Importantly, the attenuation of LV fibrosis by CD-NP treatment resulted in a preservation of diastolic and systolic function as documented by the maintenance of EF, sS, sSR, dSR-E, dSR-A, dSR-E/A. However, further studies are needed to elucidate the multiple mechanisms by which CD-NP suppresses myocardial fibrosis.

It should be noted that a limitation of the current study is the lack of BP data during the two week period of CD-NP infusion. It is possible that potential hemodynamic and vasodilating actions of CD-NP may have also accounted for some of the anti-fibrotic effects observed with this novel peptide. Further, additional studies are warranted to compare the potential anti-fibrotic effects of chronic ANP, BNP, CNP, or DNP therapy alone versus CD-NP therapy to provide supporting *in vivo* evidence that dual GC activation is superior to GC-A or GC-B activation alone. Moreover, we also need to determine the potential impact of CD-NP therapy on myocardial structure and function even in the absence of UNX.

To date, despite compelling evidence supporting a role for CNP to regulate and prevent fibrosis and extracellular matrix deposition, the therapeutic use of CNP has been limited due rapid degradation and short biological action. CD-NP may represent a therapeutic breakthrough in drug discovery since this unique peptide is not only resistant to enzymatic degradation, unlike CNP, but is also the first and only dual GC receptor agonist that currently is in clinical trials. Further, this study importantly demonstrates for the first time the potent anti-fibrotic properties of CD-NP, *in vivo*, using an experimental rat model of early cardiac fibrosis. Future *in vitro* and *in vivo* investigations are needed to assess the mechanisms by which CD-NP exerts its anti-fibrotic effects and to also assess its therapeutic properties in other models of fibrotic disease.
